# 

**Published:** 1872-01

**Authors:** 


### BOOKS AND PERIODICALS.

THE HERALD OF HEALTH is a valuable health journal, published by Wood & Holbrook, 15 Laight St., New York, at $2.00 a year.

---

GOOD HEALTH, for December has arrived, full of choice articles upon health. Alex. Moore, Publisher, Boston, at $2.00 a year.

---

THE PHILADELPHIA MEDICAL TIMES, is by far, the best printed and most ably edited medical journal that comes to our table. It is a semi-monthly journal, published by J. B. Lippencott & Co., at Philadelphia, for $4.00 a year.

THE NORTH WESTERN MEDICAL AND SURGICAL JOURNAL—edited by Alex. Stone, M. D., and published at St. Paul, Minn., at $3.00 a year, is a well edited western journal.

---

PARTURITION WITHOUT PAIN, is the title of a little book by M. L. Holbrook, M. D., just received. It is a useful little work that will interest every married lady.

---

"THE SOUTH" is the title of a beautifully printed and ably edited journal, published by Tardrew & Co., 21 Park Row, New York, at $3.00 a year.

---

PETERSON seems to become more and more popular with the ladies every year. It is only $2.00 a year, and pays elegant premiums to agents. Chas. J. Peterson, Phila., Publisher.

---

"OUR YOUNG FOLK'S ILLUSTRATED PAPER," is the title of a new eight page paper, published by E. C. Allen & Co., at Augusta, Maine, for only $1.00 a year. It is profusely illustrated.

---

AMERICAN PUBLISHER.—This is one of the best if not *the* best family newspaper that comes to our table. It is edited by Orion Clemens, brother to Mark Twain, and published at Hartford, Ct., for $1.00 a year.

---

NEW MUSICAL MAGAZINE.—"CHURCH'S MUSICAL VISITOR" for October is received. It is a new monthly devoted to Music and the Fine Arts. Published by John Church & Co., 66 West Fourth St., Cincinnati, O., at $1.00 a year.

---

THE DETROIT REVIEW OF MEDICINE AND PHARMACY, for November, is received. Geo. P. Andrews, M. D., Editor and Proprietor. A very good medical journal, and very cheap at $2.00 a year.

---

THE ALDINE — the paragon of illustrated journals. Every gentleman and lady of culture and refinement should subscribe for it. The engravings are elegant and its typography perfect. Jas. Sutton & Co., Publishers, 23 Liberty St., New York. Monthly, $2 50 a year, with chromo.

---

Any of the above journals furnished upon receipt of price, together with one copy of the BISTOURY *free.*

### SUBSCRIPTIONS RECEIVED.

In this column we will credit every subscription received during the last quarter. Subscribers will please notify us immediately if their payments are not duly credited. The State only is given, this, with the full name of the party subscribing, will be sufficient to indicate who is meant :—

NEW YORK—Mrs S P Leggett, W H Betson, R Smith, James Henry, Flander Hall, J F Hobart, A Holstead, Dr W H Hawley, G W Fisher, Miron H Clark, S M Jewett, R P Moore, L M Butterworth, K Sutt, Levi N Saunders, Alex Stephens, Dr P Wood, Dr L U Davis, Drs Stoddard & Wilson, Dr R P Upkike, Dr Ketchum, Dr Everly, Dr Sinnett, Dr M R Hawley, Dr P U Wenrich, Mrs L O Harrison, Mrs J M Slocum, Sam'l Weatherby, Nicholas Sprout, Dr Shipman, Dr B N Angell, Dr Leggett, Dr P M Stephens, Dr L Shindel, Dr V Pratt, Dr Wilcox, James A Millard, Susan P Storcum, M N Davies, Miss Sue Blake, Ben Alcott, Drs Skein & Bingham, Dr Lovejoy, Samuel Akerman, George L Whiting, Peter Bogart, Mrs S Benjamin, Mrs H A James, Dr Bahcock, Dr H Monroe, Mrs P A Lovell, Mrs John Auley, Dr. Simonson, A. L. Rodgers, H A Whitley, H Johnson, C B Mineere, B Tuthill, Sam Gridley, Ahel Jefferson, Mrs Pritchard.

PENNSYLVANIA—Pratt & Co, Mrs Mary E Crandall, John Morgan, J M Spencer, Dr W C Coleman, M Hess, Dr B F Whitmer, Mrs L A Mackey, Dr R B Watson, Dr J S K Irvin, Dr T W Meckly, Dr H H Lichtenthaler, Dr H G Smythe, A Endman, Mrs R M Bentley, Mrs Swartwood, Dr Slosson, Dr B U Wentworth, Dr S P Southerly, Dr H B Mason, Dr. Ladd, Dr Stoneman, Dr H A Snyder, Peter Bodine, Sam'l Campbell, Mrs Dixon, Miss A Weisley, Sam'l Gunterman, O Hubbell, I Samuels, X V Caulkins, A G Bucher, Dr D A Best, Dr Velder, Dr. Axtell, Dr Boyd, Mrs Cook, George Heison, Martin Quibley, Dr Spencer, A Bliven, U N Dimmock, H A Bradley, A R McKnight, Dr Dodge, Geo Ulrich, Dr Cady, Mrs Knight, John Fergison, James Fergison, John L Fergison, Michael Bunn, Dr. Storehouse, Dr Sweitzer.

ALABAMA—Dr W Stump Forwood, Dr S T Margnais, Dr P Russell.

CONNECTICUT—Mrs J Russel Dutton, Dr Ensign.

DIST, COLUMBIA—John L Allen, Mrs Martin.

FLORIDA—Dr A Michaels.

ILLINOIS—Dr W H Crane, Dr Althouse, Dr P Quibley.

INDIANA—A D Kimball, Dr Jas S Shively, Dr J S Athon, Drs Kemper & Boyden, Dr M James.

KENTUCKY—Dr Priestley, Dr S B Morgan, Mrs French.

MISSOURI—F Mitchell, Dr Pearsall.

MICHIGAN—Dr T F Dodge, Dr L R Dibble, Dr P H Joyslyn, Dr Emery, Mrs Jos Turner, Mrs P Salmon.

NEW JERSEY—Dr B F Curtiss, Dr L M Orton.

OHIO—Dr W M Shesney, Geo Sumner, A P Pollock.

WEST VIRGINIA—Dr Harden, Dr Mitchell.

WISCONSIN—G W Sheardown, Miss Priestly, Drs Sanders & French.

### A USEFUL PREMIUM.

For one hundred and ten subscribers to THE BISTOURY, at *Fifty Cents each*, amounting to $55,00, we will give a

### Grover & Baker Sewing Machine,

***Costing $55,00 !***

Everybody knows what a GROVER & BAKER Machine is, so that it requires no puffing. We will guarantee them to be new, and in perfect and complete order.

Those who prefer to work for money, can do so according to our

### CASH LIST.

Exame it and learn *How to make from ten to twenty dollars a day !*

| | | | | | |
|---|---|---|---|---|---|
| For 100 | Subscribers and | $50,00 | you may | retain | $25,00 |
| " 50 | " | 25,00 | " | " | 10,00 |
| " 25 | " | 12,50 | " | " | 5,00 |
| " 10 | " | 5,00 | " | " | 2,00 |
| " 5 | " | 2,50 | " | " | 1,00 |

So that you get your premiums in SOLID CASH, instead of cheap books, worthless pictures, or "pinched beck" jewelry.

### THE WAY TO REMIT TO US.

Collect $50 from 100 Subscribers. Then put $25 of it in your own pocket for your work, and buy a Post Office order or draft with the other $25, and remit it to us. Also, send the names and address, (Post Office, County and State,) of each subscriber, PLAINLY WRITTEN, when they will receive THE BISTOURY by return mail, which will be an evidence to them that your contract has been faithfully performed. For fifty subscribers you will send us $15, you retaining $10, and so on, down through the list.

Women, who are worn out with sewing, throw down your needles, take a copy of T-HE , nd TOURY and solicit subscriptions from your neigh bors. You will find it a healthier and more profitable employment.

Post Masters can, without trouble, secure large lists at their offices, and make it pay better than their salaries.

Young men, out of employment, can earn a suit of clothes in one day, and make their canvassing pay them ever afterwards.

Boys and girls can canvass for THE BISTOURY,

**AND MAKE MORE MONEY**

than they ever dreamed of possessing before.

Try it, everybody. There is money in it, and

**You have your Pay in your own hands.**

Send all sums over $5 by Post Office Order or Draft, made payable to THAD S. UP DE GRAFF, M. D. Smaller sums may be sent in currency.

☞. Those who desire to get up Clubs, will have sample copies sent them FREE, by writing for them. ADDRESS,

**THE BISTOURY,**

**ELMIRA, N. Y.**

